# Exploring the mechanical strength, antimicrobial performance, and bioactivity of 3D-printed silicon nitride-PEEK composites in cervical spinal cages

**DOI:** 10.36922/ijb.2124

**Published:** 2024-02-26

**Authors:** Cemile Basgul, Paul DeSantis, Tabitha Derr, Noreen J. Hickok, Ryan M. Bock, Steven M. Kurtz

**Affiliations:** 1 Implant Research Core, School of Biomedical Science, Engineering, and Health Systems, Drexel University, Philadelphia, United States of America; 2 Department of Orthopedics, Sidney Kimmel Medical College, Thomas Jefferson University, Philadelphia, United States of America; 3 SINTX Technologies, Inc., Salt Lake City, Utah, United States of America

**Keywords:** Cervical fusion cage, Anti-infection, Polyether-ether-ketone, Silicon nitride, 3D printing, ASTM F2077

## Abstract

In this study, our goal was to assess the suitability of a polyether-ether-ketone (PEEK) and silicon nitride (Si_3_N_4_) polymer composite for antimicrobial three-dimensional (3D)-printed cervical cages. Generic cage designs (PEEK and 15 vol.% Si_3_N_4_-PEEK) were 3D-printed, including solid and porous cage designs. Cages were tested in static compression, compression shear, and torsion per ASTM F2077. For antibacterial testing, virgin and composite filament samples were inoculated with *Staphylococcus epidermidis* and *Escherichia coli*. *In vitro* cell testing was conducted using MC3T3-E1 mouse preosteoblasts, where cell proliferation, cumulative mineralization, and osteogenic activity were measured. The 3D-printed PEEK and Si_3_N_4_-PEEK cages exhibited adequate mechanical strength for all designs, exceeding 14.7 kN in compression and 6.9 kN in compression shear. Si_3_N_4_-PEEK exhibited significantly lower bacterial adhesion levels, with a 93.9% reduction (1.21 log), and enhanced cell proliferation when compared to PEEK. Si_3_N_4_-PEEK would allow for custom fabrication of 3D-printed spinal implants that reduce the risk of infection compared to unfilled PEEK or metallic alloys.

## 1. Introduction

Spinal fusion is the gold-standard treatment^1^ when back pain becomes intractable, but up to 35% of patients experience failed fusions.^2–4^ These failures can result from poor osseointegration that critically depends on surface and mechanical properties of the spinal cage^5–7^ and biological factors. Among these factors, low level of bacterial contamination localized to the bone–implant interface may inhibit bone growth.^8,9^ Currently, traditionally molded or machined polyether-ether-ketone (PEEK) cages are the most common cages, which are endowed with PEEK’s strength, elastic modulus comparable to that of the bone, biocompatibility, and radiolucency.^10,11^

Earlier investigations have affirmed the robust mechanical strength of three-dimensional (3D)-printed PEEK in different implant applications.^12^ Subsequent studies have explored ways to enhance PEEK’s bioactivity by adding bioactive fillers into PEEK to allow the utilization of novel materials in 3D printing technology without compromising inherent strengths of PEEK.^13^ This pragmatic approach seeks to optimize PEEK for implants, aiming for a balanced performance that integrates strong mechanical properties with improved bioactivity.^14^ These efforts contribute to the material’s adaptability across diverse medical applications.^15^

However, enhancing cellular attraction on implant surfaces inherently increases the susceptibility to bacterial adhesion. Consequently, the imperative consideration of incorporating antimicrobial features into implant surfaces is crucial, especially when fostering osseointegration.^1,16^ Improved osteoblast adhesion and maturation have been achieved with additively manufactured solid and mesoporous PEEK materials for spinal cage applications^17,18^—currently, there are no cages that exhibit antibacterial properties. Moreover, there is a continuing unmet clinical need for biomaterials employed in spinal cages that promote osseointegration, prevent bacterial growth, withstand *in vivo* loading, and facilitate efficient medical imaging—factors that are crucial for the performance of the implant.

Among spinal cages, the ceramic silicon nitride (Si_3_N_4_), also known as as-fired-silicon-nitride (AFSN), has shown very few infections in the clinical arena.^19^ *In vitro,* Si_3_N_4_ shows decreased bacterial colonization compared to other commonly used materials and supports osteoblast maturation and mineralization.^20^ Si_3_N_4_ is radiolucent and exhibits longevity, but like all ceramic materials, it exhibits high elastic modulus, raising concerns about possible stress shielding and brittle fracture in cases where the device experiences significant non-compressive loading.^21^ To overcome these concerns, we explored the use of PEEK/Si_3_N_4_ composites. We hypothesize that this composite material will possess the osseointegrative and antimicrobial properties of Si_3_N_4_ while maintaining the mechanical properties and ductility of PEEK. Accordingly, we assessed the potential suitability of composite Si_3_N_4_-PEEK materials for fabricating 3D-printed cervical cages. Specifically, we sought to address the following questions: (i) Will 3D-printed Si_3_N_4_-PEEK cervical cages have the strength of conventional cages? (ii) Will 3D-printed Si_3_N_4_-PEEK composites exhibit antimicrobial properties? (3) How 3D-printed Si_3_N_4_-PEEK composites affect the *in vitro* cell response for osseointegration?

## 2. Methods

### 2.1. Si_3_N_4_-PEEK cages and 3D printing

**Figure 1.**
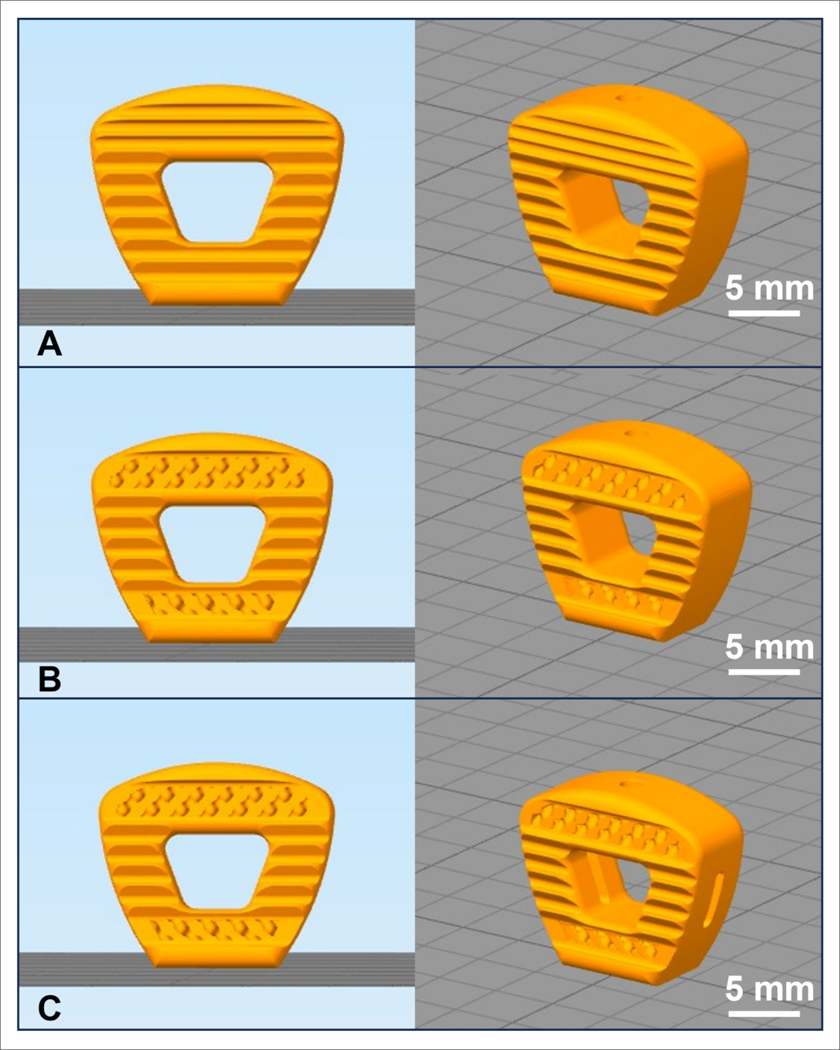
Design of 3D-printed cervical cages: solid (A), porous where top and bottom sections have porous structures (B), and porous window design with an additional window on the sides (C). Front views are shown on the left and isometric views on the right.

Cervical spinal cages utilized in this study were initially designed using Solidworks (2021, Dassault Systèmes, France) (see Figure S1 in Supplementary File). Subsequently, porous sections were designed and incorporated using nTopology (2021, Dassault Systèmes, France) with pore size ranging between 700 and 800 microns. Cages were created using fused filament fabrication (FFF) technology by a third-generation medical 3D printer (Kumovis R1, Munich, Germany) (Figure 1; Table S1 in Supplementary File). The PEEK and Si_3_N_4_-PEEK filaments (1.75 mm) used in 3D printing of cervical cages were produced by Orthoplastics (Lancashire, UK). PEEK resin was provided by Solvay (Zeniva^®^, Brussels, Belgium), and 15% volume submicron sintered β-Si_3_N_4_ powder (Flex-SN, SINTX Technologies, Salt Lake City, UT, USA) was compounded (Foster Corp., Putnam, CT, USA) with the PEEK resin to produce the composite resin used for the Si_3_N_4_-PEEK filament. The cages were printed in the upright position (Figure 1) to demonstrate the worst-case scenario, emphasizing the weakest layer adhesion for mechanical testing.

### 2.2. Mechanical testing

#### 2.2.1. Compression and compression shear

Tests were conducted on an Instron 5567 system (Instron, Norwood, MA) equipped with calibrated load and displacement sensors, with a load cell capacity of 30 kN for both compression and compression shear tests. A strain rate of 25 mm/min was chosen as per ASTM F2077,^22^ and load–displacement curves were plotted using the data (Figure S2 in Supplementary File). Stiffness values were calculated from the curves using a custom script in MATLAB 2021b, using the recommendations in ASTM F2077^22^ as a guide.

#### 2.2.2. Torsion

Prior to and after mechanical testing, each cage underwent imaging using a digital microscope (VHX-7000, Keyence). The experiments were performed utilizing an Instron 8874 system (Instron, Norwood, MA) fitted with calibrated load and displacement sensors, with a load cell capacity of 100 N∙m for torque tests. Torsion tests were carried out at 60º/min, as per the ASTM 2077^22^ recommendations, with a preloading force of 500 N applied to the cages (Figure S3 in Supplementary File).

Torque–angle curves were plotted from the data. Stiffness, yield moment, and ultimate moment values were calculated from the curves using a custom script in MATLAB 2021b, using the recommendations in ASTM F2077^22^ as a guide. To calculate the yield moment, offset angular displacement (OAD) was calculated as “3.02°” in Equation I, defined by the standard. The offset on the angular displacement axis is calculated as 10% of the intradiscal height $\left(H_{\mathrm{intradiscal}}\right)$ divided by the implant height $\left(H_{\mathrm{implant}}\right)$, multiplied by a constant.


$$OAD=\frac{H_{\mathrm{intradiscal}}\times 0.1\times 180^{{}^{\circ}}}{H_{\mathrm{implant}}\times \pi }$$

The normality of the data distributions was assessed by applying the Shapiro–Wilk test. Groups were compared via analysis of variance (ANOVA), followed by a post-hoc Tukey Honest Significant Difference Test. Furthermore, a 2 × 3 factorial ANOVA was conducted to evaluate the primary effects of material (PEEK vs. Si_3_N_4_-PEEK) and design (Solid, Porous, and Porous Window). All statistical analyses were performed with a commercially available software program (SPSS version 28), using an alpha level of 0.05.

### 2.3. Antibacterial testing

#### 2.3.1. Antibacterial adhesion

PEEK, Si_3_N_4_-PEEK, and AFSN rods (1.75 × 12 mm) were prepared, with AFSN acting as a positive control due to its demonstrated antimicrobial effect *in vivo* and it being the pure form of the antimicrobial component of the Si_3_N_4_-PEEK composite material.^21^ Rods were placed in 10% human serum, 1 × phosphate-buffered saline (PBS), and 7 mg/mL dextrose (48-well plate, *n* = 6/condition) and inoculated with 10^3^, 10^4^, or 10^5^ colony-forming units per milliliter (CFU/mL) of *Staphylococcus epidermidis* (ATCC 14990). Samples were incubated at 37°C and 95 rpm for 24 h, aseptically removed, gently rinsed 3 times in PBS, and placed in 10% trypsin^23^ to release adherent bacteria from the material surface. Serial dilutions were plated on 3M^™^ Petrifilms^™^, and colonies were then counted (countable range 20–300 CFU). Each antimicrobial experiment was independently performed 3 times and also repeated under the same conditions for *Escherichia coli* (ATCC 25922). Statistical significance was tested using a Kruskal–Wallis test (Prism v9, GraphPad, 2020).

#### 2.3.2. Scanning electron microscopy

Scanning electron microscopy (SEM) samples were prepared by aseptic removal of samples, gentle rinsing in PBS, fixing with 4% paraformaldehyde for 20 min, followed by serial dehydrations (10 min in PBS, 20% ethanol, 40% ethanol, 60% ethanol, 80% ethanol, and 100% ethanol). Samples were dried overnight, sputter-coated in 80/20 Pt/Pd, and imaged by SEM (Zeiss Supra 50VP).

### 2.4. Cell culture

PEEK, Si_3_N_4_-PEEK, AFSN, and Ti6Al4V samples (10 × 10 × 1 mm^3^) were prepared, with Ti6Al4V acting as a positive control due to its interaction with osteoblast-like cells being well-studied, and it is known to support osseointegration *in vivo.*^24^ Samples were sterilized by sonicating in 70% ethanol for 30 min, transferred to a sterile environment, soaked 3 times in 70% ethanol for 30 min, and dried overnight in the biological safety cabinet. Samples were pre-incubated for 24 h in Minimum Essential Medium α (MEM α, Gibco) supplemented with 10% fetal bovine serum (FBS; Gibco) and 1% penicillin–streptomycin (Gibco) in ultra-low attachment plates (Corning) followed by seeding with 30,000 MC3T3-E1 mouse preosteoblasts (ATCC CRL-2593); tissue culture plastic (Corning) served as a positive control. Mouse preosteoblasts were chosen because they faithfully recapitulate the molecular events that occur during osteoblast maturation and can be compared to existing work due to their immortalized nature.^25,26^ After 7 days of incubation, cell culture media were supplemented with 5 mM β-glycerophosphate (Sigma-Aldrich) and 50 μg/mL L-ascorbic acid (Sigma-Aldrich) to promote mineralization. To evaluate short-term cell attachment and proliferation, BioVision’s MTT Cell Proliferation Assay Kit was used after 24 and 72 h. To evaluate osteogenic activity, a combined approach described by Wilkesmann, Westhauser, and Fellenberg was used to simultaneously determine cell number with fluorescein-diacetate (FDA) and alkaline phosphatase activity with 4-methylumbelliferone-phosphate (4-MUP) after 7, 14, and 21 days.^27^ Mineralization was measured by staining with 2% alizarin red stain after 21 and 28 days and visualized, and then the stain was dissolved in 20% methanol and 10% acetic acid in deionized water, followed by spectrophotometric quantitation (*λ* = 405 nm, TECAN Infinite M200).

Statistical significances for each set of results were evaluated by ANOVA, followed by a post-hoc Tukey’s multiple comparisons test (*n* = 6, *α* = 0.05, Prism v9, GraphPad, 2020).

## 3. Results

### 3.1. Si_3_N_4_-PEEK cages and 3D printing

**Figure 2.**
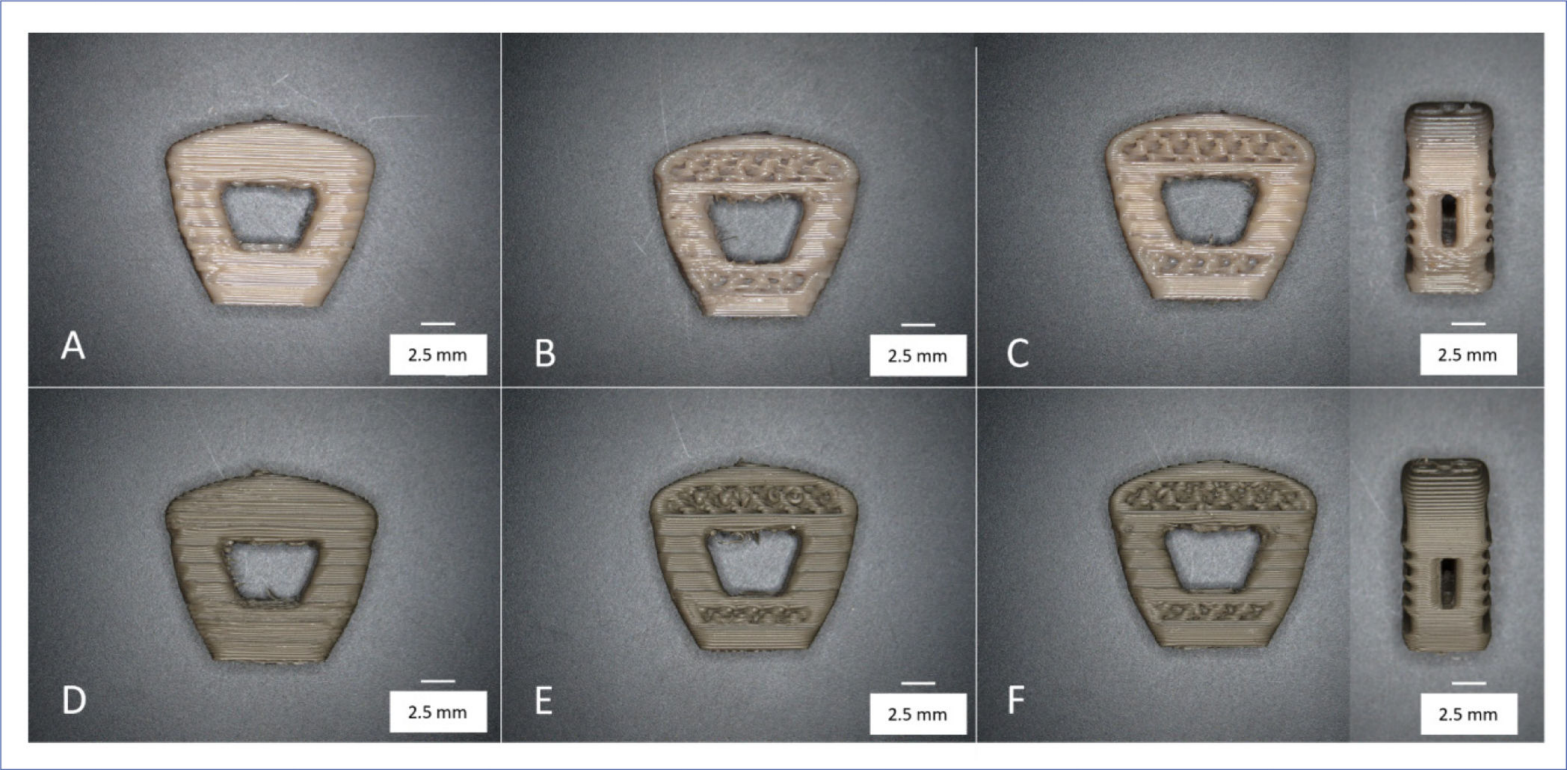
Sample cages that were 3D-printed for mechanical testing. Solid (A, D), porous (B, E), and porous window (front and side views) (C, F) designs for PEEK (A, B, C) and Si_3_N_4_-PEEK (D, E, F) materials.

A total of five cages per group of solid, porous, and porous window designs for PEEK and Si_3_N_4_-PEEK were 3D-printed and examined for structural integrity (Figure 2). Furthermore, weight measurements were conducted for each group of cages to identify outliers, addressing the possibility of any infill/extrusion issues (Table S2 in Supplementary File).

### 3.2. Mechanical testing

#### 3.2.1. Compression and compression shear

For compression, force–displacement curves of the cages that were tested above the 5th percentile ultimate force (6236 N)^28^ were plotted. For each group, the first linear regions were defined separately, and the stiffness (Table 1) was computed by determining the slope of the linear region for each sample.

The data demonstrated a normal distribution for the majority of the groups (four out of six). According to 2 × 3 factorial ANOVA, design was significantly affecting the stiffness under compression (*p* < 0.001). The stiffness of the porous design was significantly higher than the solid design (mean difference = 6185 N/mm, *p* < 0.001) and the porous window design (mean difference = 1606 N/mm, *p* = 0.01). The stiffness of the porous window design was also significantly higher than the solid design (mean difference = 4579 N/mm, *p* < 0.001). When stiffness was examined as a function of material, the main effect was not significant in stiffness of the cages as well as the interaction between material and design.

**Figure 3.**
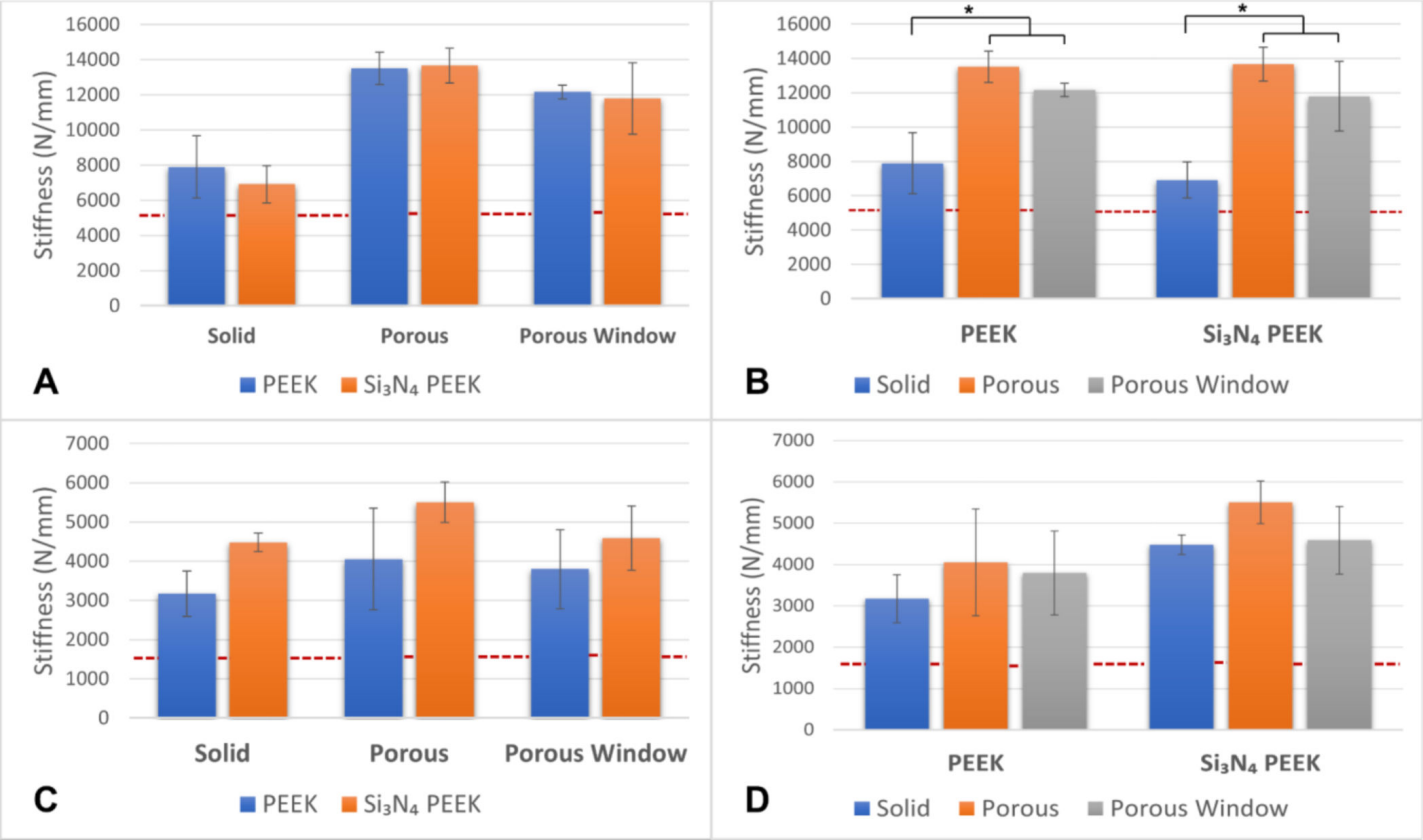
Comparison in stiffness between PEEK and Si_3_N_4_-PEEK per solid, porous, and porous window designs: compression (A, B) and compression shear (C, D). Dotted line indicates the 5th percentile stiffness as per Peck et al.^28^ without shear in the beginning.

In addition to the main effects, the group comparisons between PEEK and Si_3_N_4_-PEEK and the designs were analyzed (Figure 3).

There was no significant difference in stiffness between PEEK and Si_3_N_4_-PEEK per design (Figure 3A). The compression stiffness of PEEK porous and porous window designs was significantly higher than that of the solid design (mean difference = 5622 and 4277 N/mm respectively, *p* < 0.001 for both). Similarly, for Si_3_N_4_-PEEK, porous and porous window designs achieved higher compression stiffness than solid design (mean difference = 6748 and 4881 N/mm respectively, *p* < 0.001 for both) (Figure 3B). In addition to surpassing the 5th percentile of ultimate strength loading,^28^ porous window designs for both PEEK and Si_3_N_4_-PEEK materials were tested above 50th percentile of ultimate compression strength defined by Peck et al.^28^ (10,800 N) (Table S3 in Supplementary File). Furthermore, solid and porous designs for both PEEK and Si_3_N_4_-PEEK materials were tested above 75th percentile of ultimate compression strength defined by Peck et al.^28^ (14,728 N) (Table S3 in Supplementary File).

For compression shear tests, the cages were tested above the 5th percentile ultimate shear force (1515 N).^28^ The force–displacement curves were plotted for each sample. Data were normally distributed for all groups. According to 2 × 3 factorial ANOVA, the main effect of material was significantly affecting the shear stiffness (*p* < 0.001). Si_3_N_4_-PEEK cages had significantly higher stiffness than PEEK cages (mean difference = 1185 N/mm, *p* < 0.001). The main effect of the design was observed within the borderline significance level (*p* = 0.049). The porous design’s shear stiffness was slightly higher than the solid design’s stiffness (mean difference = 950 N/mm, *p* = 0.04). Finally, the interaction between the main effects (material and design) was not significant.

In addition to the main effects, the group comparisons between PEEK and Si_3_N_4_-PEEK and the designs were analyzed (Figure 3). There was no significant difference in shear stiffness between PEEK and Si_3_N_4_-PEEK per design (Figure 3C). Similarly, the shear stiffness between the designs for both PEEK and Si_3_N_4_-PEEK was not significantly different (Figure 3D). In addition to 5th percentile of ultimate shear strength loading,^28^ porous window designs for PEEK were tested above 50th percentile of ultimate shear strength defined by Peck et al.^28^ (4626 N) (Table S3 in Supplementary File). Furthermore, solid and porous designs for both PEEK and Si_3_N_4_-PEEK materials and porous window design of Si_3_N_4_-PEEK were tested above 75th percentile of ultimate shear strength defined by Peck et al. (6868 N) (Table S3 in Supplementary File).

#### 3.2.2. Torsion

**Figure 4.**
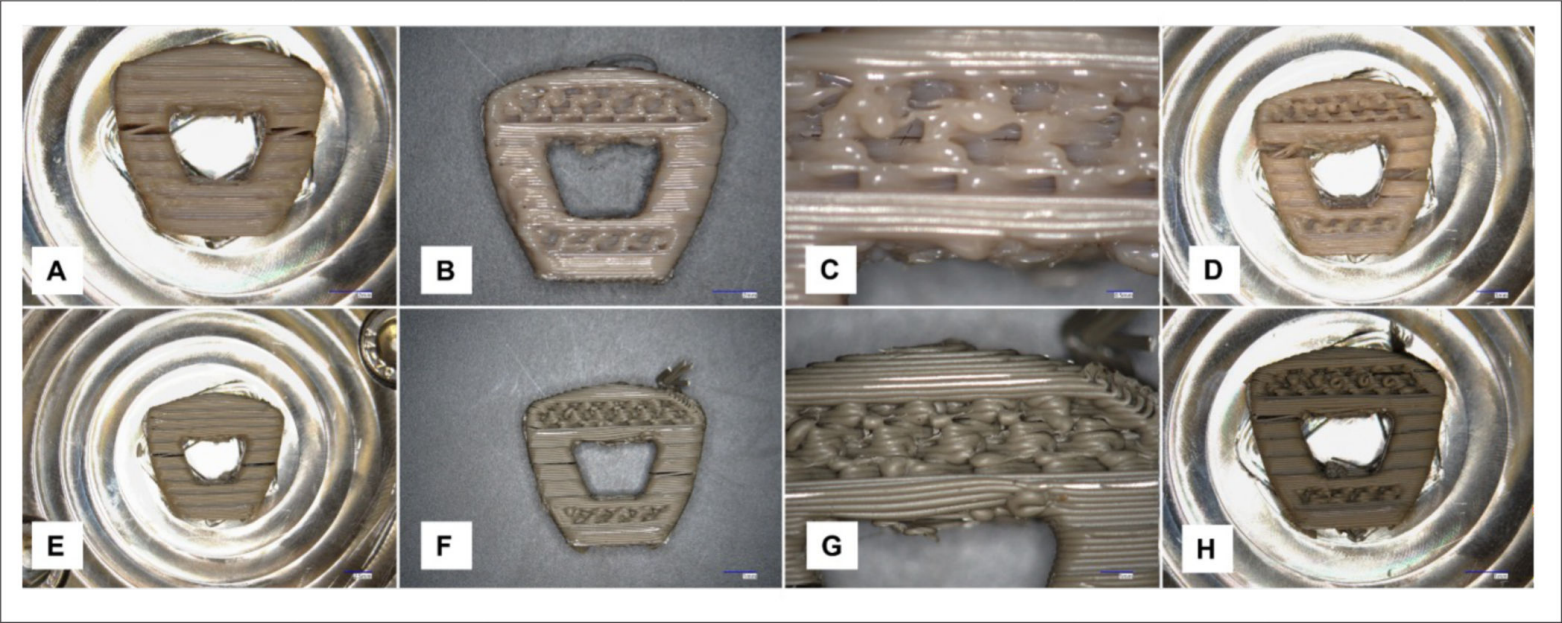
Samples after torsion testing: PEEK solid (A), porous (B, C), and porous window (D) designs, Si_3_N_4_-PEEK solid (E), porous (F, G), and porous window (H) designs. Close-up views of porous sections are shown in (C) and (G).

For torsion tests, the cages were tested until failure according to ASTM F2077^22^ (Figure 4).

The torque–angle curves were plotted for each sample. For each sample, ultimate moment, yield moment, and stiffness values were calculated from the graphs (Table 1).

Torsional stiffness data were normally distributed for all the groups. According to 2 × 3 factorial ANOVA, both main effects (material and design) significantly affected the torsional stiffness of cages (*p* = 0.001 and *p* < 0.001, respectively). Si_3_N_4_-PEEK cages had significantly higher torsional stiffness than PEEK cages (mean difference = 0.10 Nm/deg, *p* = 0.001). Cages with the solid design achieved the highest torsional stiffness and that was significantly higher than the cages with the porous and porous window design (mean difference = 0.13 and 0.14 Nm/deg, *p* = 0.004 and *p* = 0.002, respectively). Finally, the interaction between the main effects (material and design) was not significant.

**Figure 5.**
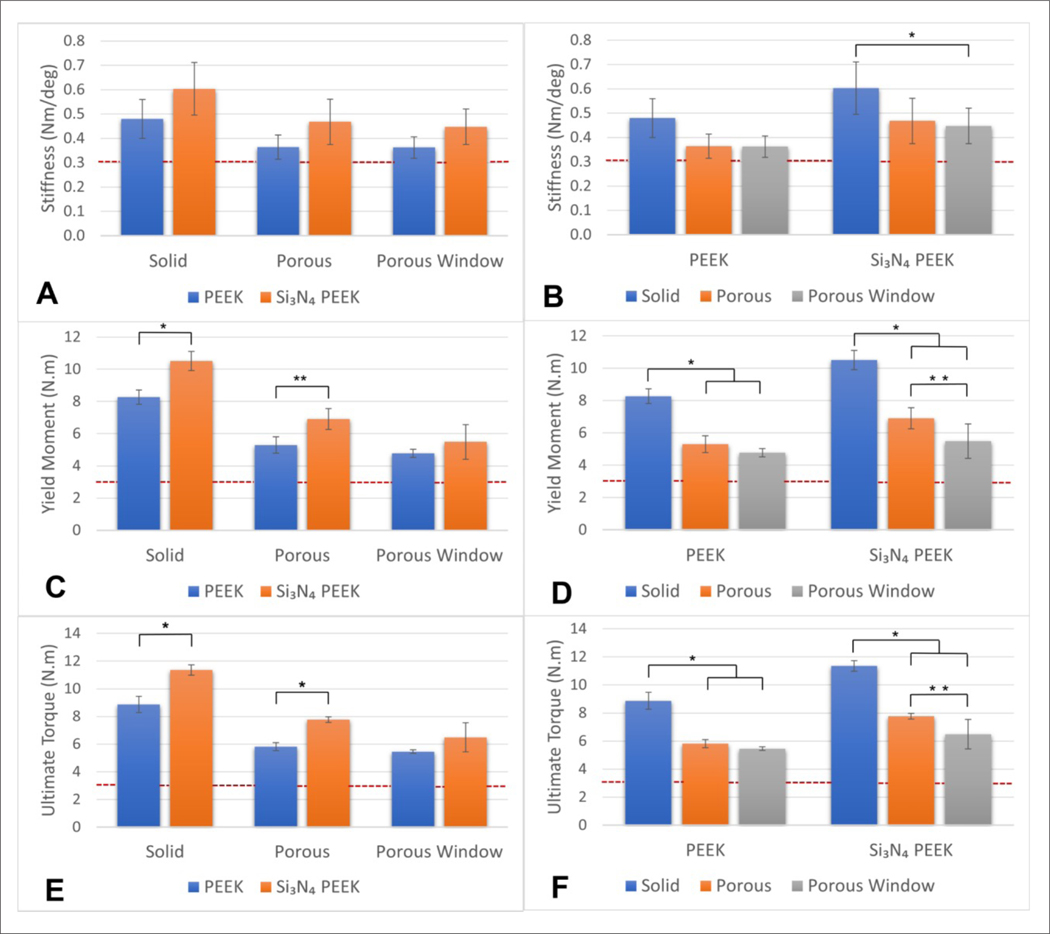
Comparison between PEEK and Si_3_N_4_-PEEK designs: torsional stiffness (A, B), yield moment (C, D), and ultimate torque (E, F). (A, C, E) Comparison based on the material. (B, D, F) Comparison based on the designs across the same material. Dotted line indicates the 5th percentile as per Peck et al.^28^

In addition to the main effects, the group comparisons between PEEK and Si_3_N_4_-PEEK and the designs were analyzed. The difference between PEEK and Si_3_N_4_-PEEK cages’ stiffness was not significant per design (Figure 5A). In the comparison of designs, it was observed that the stiffness of Si_3_N_4_-PEEK solid cages was slightly greater than that of Si_3_N_4_-PEEK porous window cages (mean difference = 0.16 Nm/deg and *p* = 0.04) (Figure 5B).

Yield moment data were normally distributed for all the groups. According to 2 × 3 factorial ANOVA, both main effects (material and design) significantly affected the yield moment of cages (*p* = 0.001 and *p* < 0.001, respectively). Si_3_N_4_-PEEK cages had significantly higher yield moment than PEEK cages (mean difference = 1.52 N·m, *p* < 0.001). Cages with the solid design achieved the highest yield moment and that was significantly higher than that of the cages with porous and porous window design (mean difference = 3.29 and 4.26 N·m, respectively, *p* < 0.001 for both). In addition, porous design’s yield torque was significantly higher than the porous window design (mean difference = 0.97 N·m, *p* = 0.002). Finally, the interaction between the main effects (material and design) was small but significant (*p* = 0.04).

In addition to the main effects, the group comparisons between PEEK and Si_3_N_4_-PEEK and their designs were analyzed (Figure 5C and D). Si_3_N_4_-PEEK cages demonstrated higher yield moment than PEEK cages both for solid and porous designs (mean difference = 2.24 and 1.61, *p* < 0.001 and *p* = 0.007, respectively) (Figure 5C). For PEEK, solid cages, yield moment was higher than both porous and porous window cages (mean difference = 2.97 and 3.49 N·m, respectively, *p* < 0.001). Similarly, Si_3_N_4_-PEEK solid cages demonstrated higher yield torque than both Si_3_N_4_-PEEK porous and porous window cages (mean difference = 3.60 and 5.02, respectively, *p* < 0.001). Additionally, porous window design had lower yield torque compared to porous design of Si_3_N_4_-PEEK cages (mean difference = 1.42 N·m, *p* = 0.02) (Figure 5D).

Ultimate moment data were normally distributed for the majority of the groups (five out of six). According to 2 × 3 factorial ANOVA, both main effects (material and design) significantly affected the ultimate moment of cages (*p* < 0.001). Si_3_N_4_-PEEK cages had significantly higher ultimate moment than PEEK cages (mean difference = 1.83 N·m, *p* < 0.001). Cages with the solid design achieved the highest ultimate torque and that was significantly higher than that of the cages with the porous and porous window design (mean difference = 3.32 and 4.14 N·m respectively, *p* < 0.001 for both). In addition, porous design’s ultimate torque was significantly higher than that of the porous window design (mean difference = 0.82 N·m, *p* = 0.002). Finally, the interaction between the main effects (material and design) was small but significant (*p* = 0.02).

In addition to the main effects, the group comparisons between PEEK and Si_3_N_4_-PEEK and the designs were analyzed (Figure 5E and F). Si_3_N_4_-PEEK cages of both solid and porous designs showed significantly higher ultimate moment than PEEK cages (mean difference = 2.49 and 1.95 N·m, *p* < 0.001) (Figure 5E). For PEEK, solid cages’ ultimate moment was higher than both porous and porous window design cages (mean difference = 3.05 and 3.41, respectively, *p* < 0.001 for both). Similarly, Si_3_N_4_-PEEK solid cages demonstrated higher ultimate torque than both Si_3_N_4_-PEEK porous and porous window cages (mean difference = 3.59 and 4.87, respectively, *p* < 0.001 for both). Additionally, porous window design caused lower ultimate torque compared to porous design for Si_3_N_4_-PEEK cages (mean difference = 1.26 Nm, *p* = 0.01) (Figure 5F).

### 3.3. Antibacterial testing

**Figure 6.**
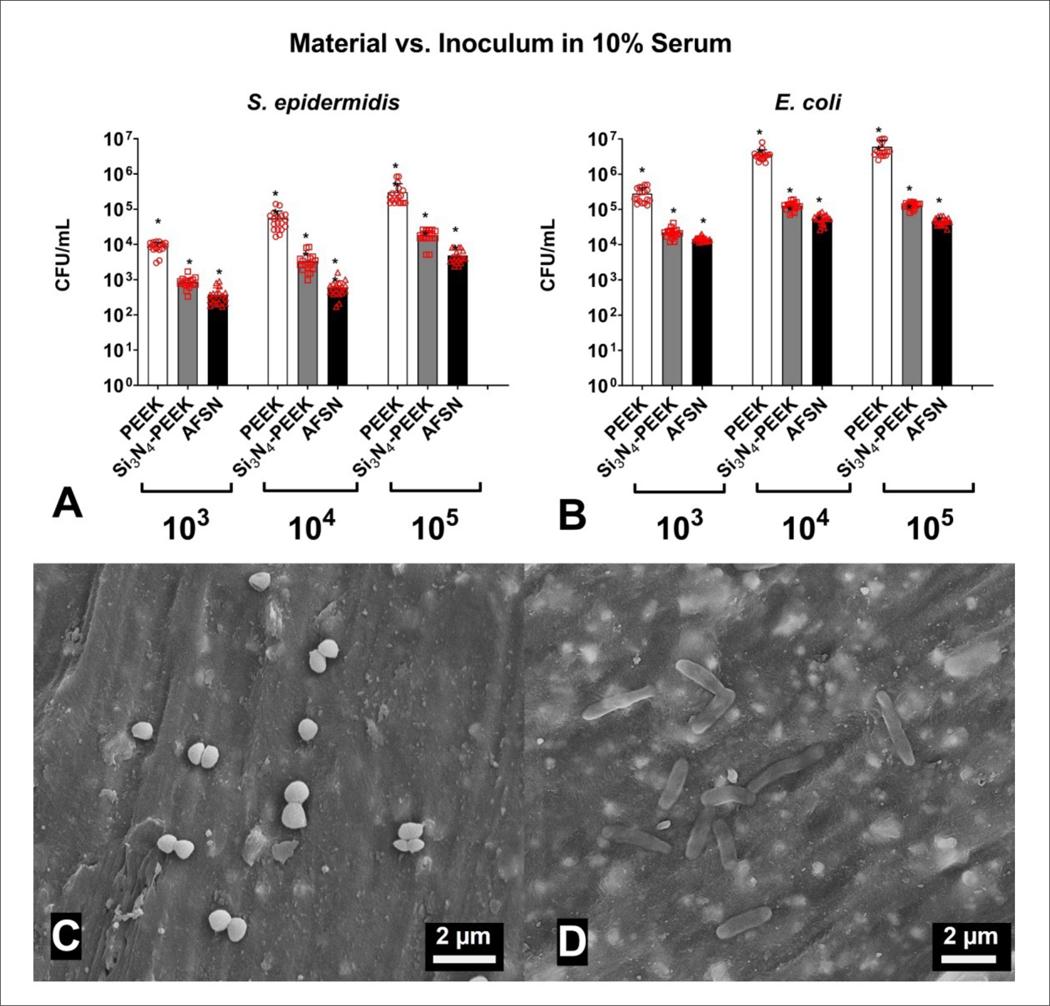
Effects of inoculum and material on bacterial colonization. Number of colony-forming units per milliliter for *S. epidermidis* (A) and *E. coli* (B) for PEEK, Si_3_N_4_-PEEK, and AFSN materials. Scanning electron micrographs of *S. epidermidis* (a gram-positive bacterium that grows in grape-like clusters) (C) and *E. coli* (a gram-negative bacterium with a rod-like structure) (D) on Si_3_N_4_-PEEK samples after 24 h.

Because Si_3_N_4_ has been reported to possess antibacterial activity, we tested the ability of the different materials to inhibit bacterial adhesion, expressed as colony-forming units per milliliter (CFU/mL). For each level of inoculum and for both *S. epidermidis* and *E. coli*, AFSN samples consistently had the lowest numbers of adherent bacteria, indicating the greatest overall antimicrobial effect (Figure 6). Si_3_N_4_-PEEK consistently showed greater antibacterial activity than pure PEEK samples (*p* < 0.05), but less antibacterial activity when compared to pure Si_3_N_4_ (*p* < 0.05).

When incubated with *S. epidermidis*, Si_3_N_4_-PEEK samples showed a 1.02, 1.21, and 1.22 log reduction for starting inocula of 10^3^, 10^4^, and 10^5^ CFU/mL, respectively, as compared to PEEK samples. For *E. coli*, mean log reductions were 1.10, 1.45, and 1.66. When all groups were pooled, the average mean log reduction for Si_3_N_4_-PEEK compared to PEEK was 1.28. Representative SEM micrographs of *S. epidermidis* and *E. coli* on Si_3_N_4_-PEEK samples are shown in Figure 6C and D.

### 3.4. Osteogenic activity of cells cultured on different substrates

**Figure 7.**
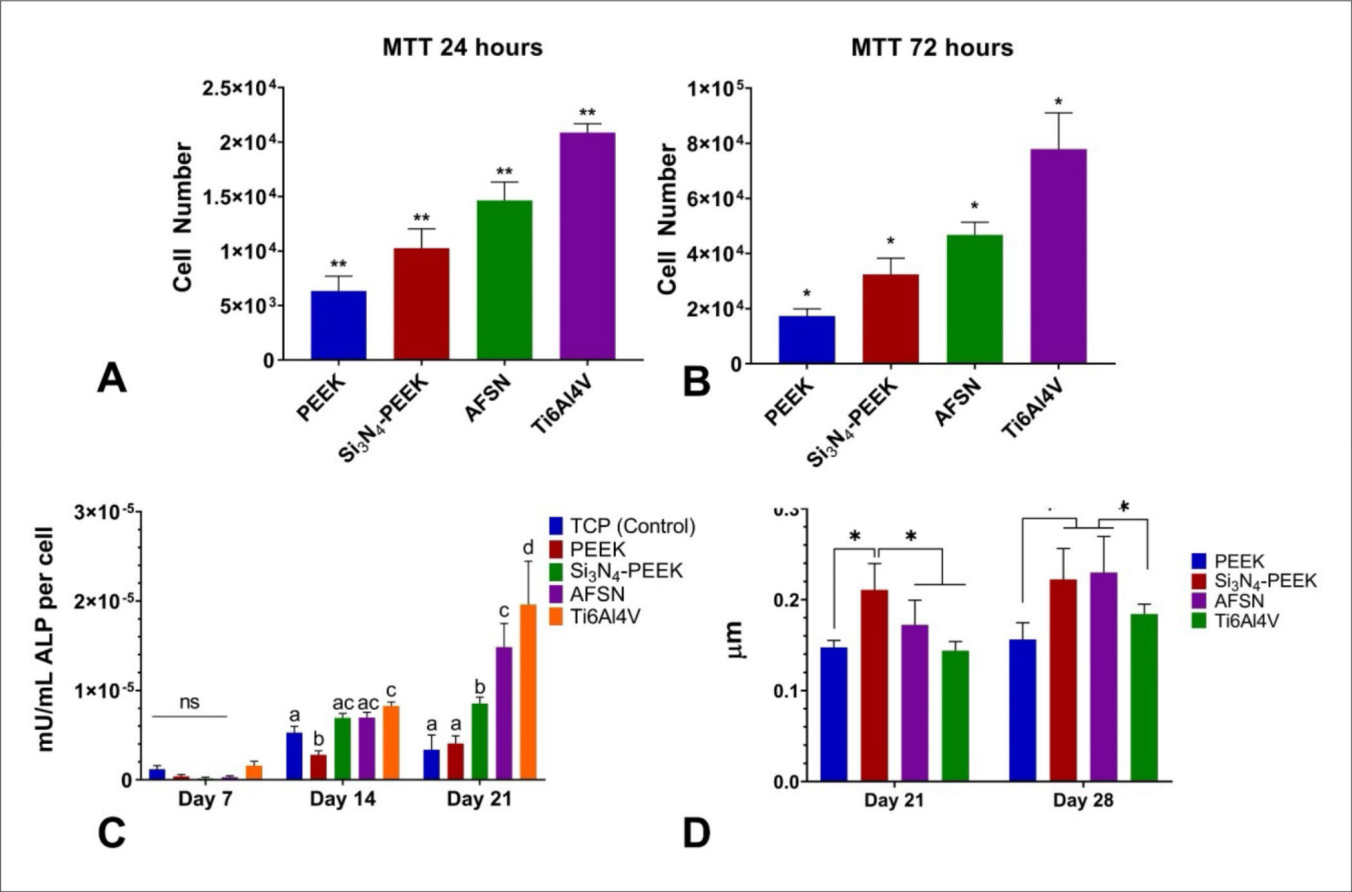
Comparison of cell number via MTT assay at 24 h (A) and 72 h (B) on PEEK, Si_3_N_4_-PEEK (SN-PEEK), as-fired silicon nitride (AFSN), and Titanium (Ti) alloy (Ti6Al4V). (C) Comparison of normalized osteogenic activity between control group, PEEK, Si_3_N_4_-PEEK, AFSN, and Ti for each time point; groups sharing the same letter are not significantly different from each other (*p* < 0.05). (D) Cumulative mineralization on PEEK, Si_3_N_4_-PEEK, AFSN, and Ti surfaces at days 21 and 28.

MTT assay was used to quantify short-term cell proliferation of the mouse preosteoblasts on each surface (Figure 7). Absorbance results for each group were normalized to a known number of cells to determine the approximate number of cells on each surface. The number of cells for each group was significantly different from each other at both 24 h (*p* < 0.01) and 72 h (*p* < 0.05). At both time points, Si_3_N_4_-PEEK surfaces showed higher cell proliferation when compared to PEEK and AFSN (Figure 7A and B). Ti6Al4V (control group), a titanium alloy commonly used in orthopedic implants, had the highest average number of cells overall at both time points.

Normalized osteogenic activity was determined by dividing the alkaline phosphatase activity determined with 4-MUP, by cell number determined by an FDA stain assay. After 7 days, there was no significant difference in osteogenic activity between any surfaces; however, this result was expected due to the mineralization media not being introduced until after this time point. On day 14, the PEEK group had significantly less osteogenic activity when compared to all other groups at this time point, and Si_3_N_4_-PEEK performed similarly to AFSN. The Ti group had the highest overall average osteogenic activity at this time point. On day 21, PEEK was not significantly different from tissue culture plastic (TCP), the group with the lowest average normalized osteogenic activity. Normalized osteogenic activity was significantly increased for Si_3_N_4_-PEEK when compared to PEEK, and significantly increased for AFSN when compared to Si_3_N_4_-PEEK. The Ti had significantly more osteogenic activity than all other groups at this time point (Figure 7C).

Alizarin Red binds to calcium and can be used to quantify cumulative mineralization of cell culture samples. After 21 days, Si_3_N_4_-PEEK had significantly more Alizarin Red stain than all other groups (*p* < 0.05), indicating the highest amount of cumulative mineralization. After 28 days, there was no significant difference between the Si_3_N_4_-PEEK and AFSN groups, but both of these groups had significantly more Alizarin Red stain than the PEEK and Ti groups (*p* < 0.05) (Figure 7D).

## 4. Discussion

Infection represents a significant risk factor contributing to spinal implant failures, underscoring the critical need for biomedical materials possessing not only excellent mechanical and pro-osteogenic characteristics but also robust antimicrobial properties. A composite material comprising Si_3_N_4_-PEEK holds the potential to allow fabrication of customized 3D-printed spinal implants, with the additional benefit of being mildly antimicrobial, a property that contemporary implant materials like unfilled PEEK and metal alloys do not possess. Mechanically, the 3D-printed PEEK and Si_3_N_4_-PEEK cages exhibited a gradual yielding behavior across all designs. Notably, the mean strength of the generic 3D-printed cage designs exceeded the 75th percentile benchmarks established by Peck et al.,^28^ registering at 14.7 kN in compression and 6.9 kN in compression shear. In terms of biological activity, the Si_3_N_4_-PEEK material demonstrated a 93.9% reduction (~1.2 log or 12-fold) in bacterial adhesion when compared to PEEK (*p* < 0.01). Significantly higher cell proliferation was observed on Si_3_N_4_-PEEK as compared with PEEK, indicating its bio- and osteocompatibility of Si_3_N_4_-PEEK. In keeping with osteocompatibility, the cumulative mineralization of cell culture samples was higher on Si_3_N_4_-PEEK samples than on PEEK.

Several limitations to the current study should be recognized. The mechanical results obtained in this study are linked to the specific setup of the printer and the consistent temperature conditions utilized during the research. It is reasonable to anticipate that alterations in the printer’s setup or adjustments in its configurations, as well as variations in temperature conditions, could lead to different effects on the mechanical properties and porosity of 3D-printed PEEK cages, which might differ from those observed in this study. The incorporation of Si_3_N_4_ into PEEK was constrained by the extrusion capabilities and filament condition required for successful fused filament fabrication of the cages and specimens utilized in this study. Another limitation was that our antibacterial testing concentrated on *S. epidermidis* and *E. coli*, which might not encompass the full spectrum of potentially relevant pathogens. Furthermore, although we assessed cell proliferation and osteogenic activity using mouse preosteoblasts, these cells show only weak osteoblastic cell activity, and the interactions will best be determined in studies that place the implants into bone defects.

Previously, Peck et al.^28^ investigated the mechanical performance of cervical cages that were submitted to the Food and Drug Administration (FDA), and they included unique materials other than metal and PEEK. Using these data as benchmarks, our results showed that all the designs with both materials (Si_3_N_4_-PEEK and PEEK) achieved more than 5th percentile stiffness under all three forces. In addition, cages were tested above 50th percentile of ultimate compression and compression shear strength defined by Peck et al.^28^ Furthermore, the cervical cages examined in this study surpassed 104% of the ultimate load capacity in compression and exhibited over 3 times the shear strength compared to machined cages previously reported in the literature.^17^ While the primary goal of incorporating Si_3_N_4_ into PEEK was to enhance its antibacterial properties, it was notable that this process resulted in an increased torsional strength of the cages.

Contrastingly, the stiffness of solid cages was found to be lower than that of porous designs under compression and shear. This unexpected result may be attributed to varying loading mechanisms, which necessitate testing in compression, shear, and torsion, as mandated by ASTM standards for cages.^22^ The current study focused on the worst-case scenario, emphasizing the critical role of layer bonding in interpreting results. The lower stiffness observed in solid cages under compression and shear forces was unforeseen. The 3D printer used in the study employed an additional cooling setting, partially activated to facilitate inter-layer cooling for subsequent layers. Excessive cooling, however, could compromise layer adhesion. The porous sections, having less material and shorter cooling times, may have exhibited better adherence under similar cooling conditions than their solid counterparts. This phenomenon is less pronounced in torsion testing, where the unique loading conditions involve layer compression up to 500 N, followed by layer twisting, favoring solid layers. This underscores the significance of carefully controlling printing conditions when working with PEEK implants.

There exist differing opinions regarding the incorporation of ceramics into PEEK. Some studies suggest an increase^29,30^ while others have demonstrated a decrease in strength.^31,32^ Research indicating increased strength implies the ability of polymer–ceramic matrix to endure higher loads, effectively transferring some stress from polymer to ceramic and leveraging on the ceramic’s higher strength.^33^ Conversely, discussions on strength reduction highlight concerns about the limited interfacial interaction between the polymer and ceramic.^34^ It is crucial to acknowledge that factors such as volume content, microstructure, chemical composition, ceramic properties, and the bonding between PEEK and ceramic play pivotal roles in simultaneously enhancing mechanical strength.^29^ In our study, Si_3_N_4_-PEEK cages showed higher torsional strength than PEEK cages. Regarding the designs, solid cages achieved the highest torsional strength, and porous cages were stronger than porous cages with windows. In a similar manner, Fogel et al.^35^ investigated the design influence on mechanical performance of spinal cages and indicated higher stiffness for solid design.

Previous studies investigated Si_3_N_4_^19,20,36^ and Si_3_N_4_-PEEK^37–39^ for their ability to enhance the biological activities of PEEK-based implants—including maturation of osteoblasts and antimicrobial activity.^20,39^ Gorth et al. found decreased biofilm formation as well as fewer live bacteria on both the as-fired (AFSN) and polished Si_3_N_4_ compared with PEEK and titanium surfaces.^20^ In a companion *in vivo* study, Webster et al.^19^ observed improved osseointegration for Si_3_N_4_ samples relative to PEEK and Ti6Al4V implanted into rat calvaria wound sites contaminated with *S. epidermidis*. Bock et al.^36^ showed similar results *in vitro* using a human plasma-based inoculum with *S. epidermidis* and *E. coli* exposed to a range of surface-modified Si_3_N_4_ materials. Pezzotti et al.^37^ found that incorporating 15 vol.% coarse (approx. 50–250 μm) Si_3_N_4_ into PEEK led to improved proliferation and mineralization of SaOS2 cells in addition to a 1-log_10_ reduction in adherent *S. epidermidis* relative to monolithic PEEK following a 24-h exposure *in vitro*. Marin et al.^38^ observed increased alkaline phosphatase activity and mineralization of KUSA-A1 mesenchymal stem cells exposed to the same coarse, 15 vol.% Si_3_N_4_-PEEK composite relative to monolithic PEEK, but cell proliferation was not improved. It was hypothesized that the large regions of PEEK between the coarse silicon nitride particles were responsible for this lack of improvement since Si_3_N_4_ is thought to act at short distances, very near its surface, through the products of hydrolysis reactions. This observation, along with 3D printing requirements, led to the modification of the powder feedstock used for the composite in this study to obtain a submicron size distribution. Hu et al.^39^ demonstrated the potential of Si_3_N_4_-PEEK in biomedical applications as it exhibited osteogenic and antibacterial activities. Our findings clearly showed that a 3D-printed Si_3_N_4_-PEEK composite was able to achieve a significant reduction in numbers of adherent bacteria, with slightly greater activity against gram-negative bacteria. This latter finding is of importance as these microorganisms are the most common causes of deep infections.^40^ It is probable that increased antimicrobial properties could be achieved with higher concentrations of Si_3_N_4_, but these increases will need to be balanced with the composite material’s ability to be 3D-printed and the effects on mechanical properties as the Si_3_N_4_ concentration probes or exceeds the percolation threshold.

In addition to reduced bacteria activity, Si_3_N_4_ coating onto PEEK surfaces was shown to promote cell responses *in vitro* and improve osseointegration *in vivo.*^41,42^ The application of Si_3_N_4_ coating led to increased adhesion, proliferation, differentiation, and osteoblast gene expression using MC3T3-E1 cells *in vitro*. Moreover, the bioactive Si_3_N_4_ coating on PEEK facilitated bone regeneration and enhanced osseointegration *in vivo.*^41^ Hu et al.^42^ found that Si_3_N_4_-coated PEEK significantly enhanced the adhesion, proliferation, differentiation, and expression of osteogenesis-related genes in rat bone marrow stromal cells (rBMSCs) when compared to PEEK. In our research, Si_3_N_4_-PEEK enhanced cell proliferation and also increased the normalized osteogenic activity in comparison to PEEK; AFSN showed greater proliferation and osteogenic maturation than either Si_3_N_4_-PEEK or PEEK itself. Importantly, cumulative mineralization at 28 days showed no differences between the Si_3_N_4_-PEEK and AFSN groups, and perhaps more importantly, both were greater than that measured for the PEEK and Ti6Al4V groups.

## 5. Conclusion

Various designs of Si_3_N_4_-PEEK spinal cages fabricated using fused filament fabrication were assessed for mechanical strength. The findings revealed that Si_3_N_4_-PEEK cages exhibited satisfactory mechanical strength across all designs tested in this study. Further, the Si_3_N_4_ additive concentration was sufficiently low to maintain the plastic properties of the PEEK matrix. Antimicrobial activity and osseocompatibility were compared on Si_3_N_4_-PEEK with virgin PEEK, AFSN, and titanium surfaces. Compared to PEEK, Si_3_N_4_-PEEK surfaces demonstrated reduced bacterial adhesion, and increased osteoblast-like cell proliferation and mineralization. These results suggest that Si_3_N_4_-PEEK holds promise as a viable biomaterial for spinal implant applications.

## Supplementary Material

Supplementary Material

## Figures and Tables

**Table 1.** Mechanical testing metrics calculated for cages

	PEEK solid	PEEK porous	PEEK porous window	Si_3_N_4_-PEEK solid	Si_3_N_4_-PEEK porous	Si_3_N_4_-PEEK porous window
Compression stiffness (N/mm)	7886 ± 1771	13508 ± 918	12163 ± 388	6911 ± 1053	13659 ± 997	11791 ± 2032
Shear stiffness (N/mm)	3171 ± 580	4052 ± 1291	3793 ± 1011	4482 ± 235	5501 ± 517	4587 ± 816
Torsional stiffness (Nm/deg)	0.48 ± 0.08	0.36 ± 0.05	0.36 ± 0.04	0.60 ± 0.11	0.47 ± 0.09	0.45 ± 0.07
Yield moment (N∙m)	8.27 ± 0.45	5.29 ± 0.52	4.77 ± 0.26	10.5 ± 0.60	6.90 ± 0.65	5.49 ± 1.07
Ultimate moment (N∙m)	8.86 ± 0.60	5.82 ± 0.28	5.45 ± 0.13	11.4 ± 0.37	7.76 ± 0.21	6.49 ± 1.05

## Data Availability

Data are available from the corresponding author upon reasonable request.
