# Supplementary material for: Exploring the mechanical strength, antimicrobial performance, and bioactivity of 3D-printed silicon nitride-PEEK composites in cervical spinal cages
Source: Int J Bioprint. Author manuscript; Available in PMC 2025 Sep 4. (PMC12406975; doi:10.36922/ijb.2124)
Supplement: Supplementary Material [file NIHMS2099046-supplement-Supplementary_Material.pdf]

ACCSCIENCE PUBLISHING

International Journal of Bioprinting

RESEARCH ARTICLE

Exploring the mechanical strength, antimicrobial performance, and bioactivity of 3D-printed silicon nitride-PEEK composites in cervical spinal cages

Supplementary File

Table S1. Printing parameters used in this study to 3D-print cervical spinal cages

	PEEK	Si_3N_4-PEEK
Nozzle diameter (mm)	0.4	
Extrusion multiplier	1.01	0.96–1.2
Extruder width (mm)	0.45	
Layer height (mm)	0.18	
Bed temperature (°C)	250	
Nozzle temperature (°C)	420	425
Chamber temperature (°C)	200	
Z-axis movement speed (mm/min)	500	
Interior fill percentage (%)	100	

Table S2. Representative weight measurements of cages printed for study groups

Material	Design	Weight (g)
**PEEK**	Solid	1.14
**PEEK**	Solid	1.15
**PEEK**	Solid	1.17
**PEEK**	Porous	0.96
**PEEK**	Porous	0.92
**PEEK**	Porous	0.95
**PEEK**	Porous window	0.9
**PEEK**	Porous window	0.91
**PEEK**	Porous window	0.92
**Si_3N_4-PEEK**	Solid	1.38
**Si_3N_4-PEEK**	Solid	1.36
**Si_3N_4-PEEK**	Solid	1.42
**Si_3N_4-PEEK**	Solid	1.44
**Si_3N_4-PEEK**	Solid	1.27
**Si_3N_4-PEEK**	Solid	1.36
**Si_3N_4-PEEK**	Porous	1.29
**Si_3N_4-PEEK**	Porous	1.3
**Si_3N_4-PEEK**	Porous	1.31

(Continued...)

Table S2. *Continued...*

**Si_3N_4-PEEK**	Porous	1.12
**Si_3N_4-PEEK**	Porous	1.3
**Si_3N_4-PEEK**	Porous	1.14
**Si_3N_4-PEEK**	Porous window	1.08
**Si_3N_4-PEEK**	Porous window	1.13
**Si_3N_4-PEEK**	Porous window	1.13
**Si_3N_4-PEEK**	Porous window	1.13
**Si_3N_4-PEEK**	Porous window	1.03
**Si_3N_4-PEEK**	Porous window	1.12

Table S3. Cage testing conditions for different tests conducted in this study

**Design and material/ mechanical test**	**Solid**		**Porous**		**Porous window**	
	**PEEK**	**Si_3N_4-PEEK**	**PEEK**	**Si_3N_4-PEEK**	**PEEK**	**Si_3N_4-PEEK**
**Compression**	5th, 50th, 75th*		5th, 50th, 75th*		5th, 50th*	
**Compression shear**	5th, 50th, 75th*		5th, 50th, 75th*		5th, 50th*	5th, 50th, 75th*
**Torsion**	until failure					

*Percentiles were based on Peck et al. study.[1]

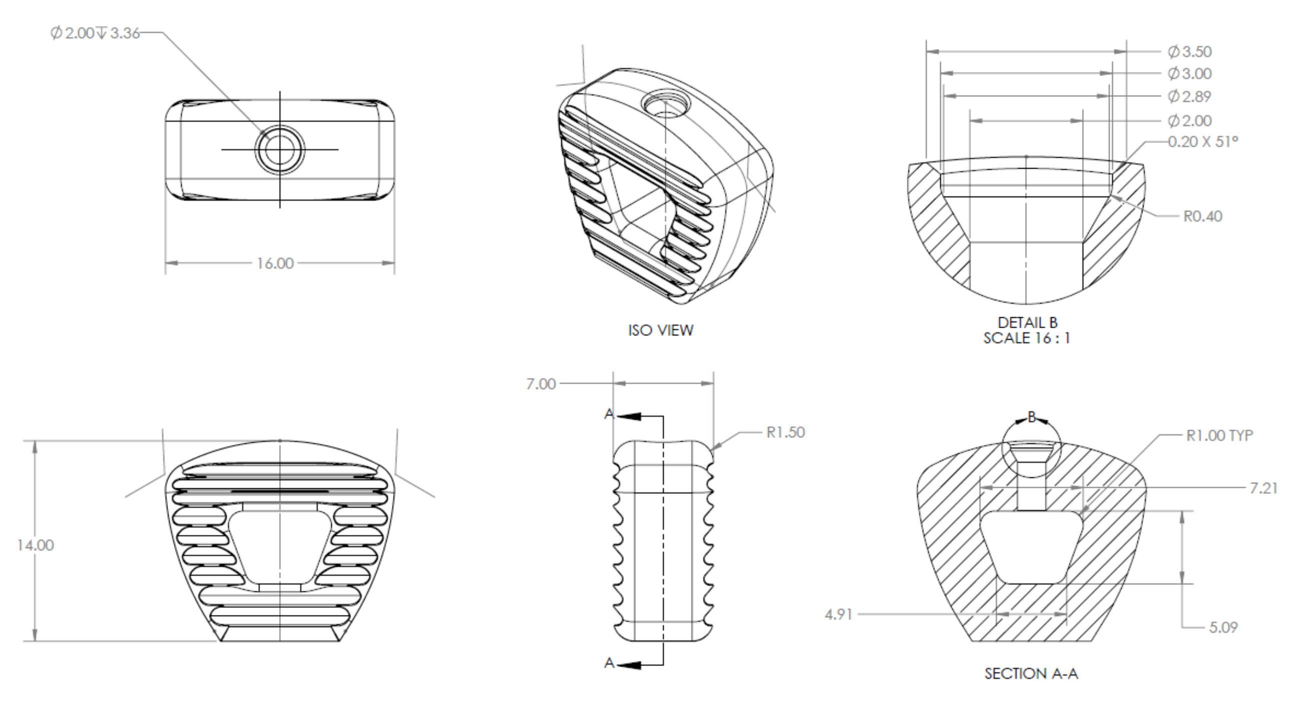

Figure S1. Dimensions of the solid cage design used in this study.

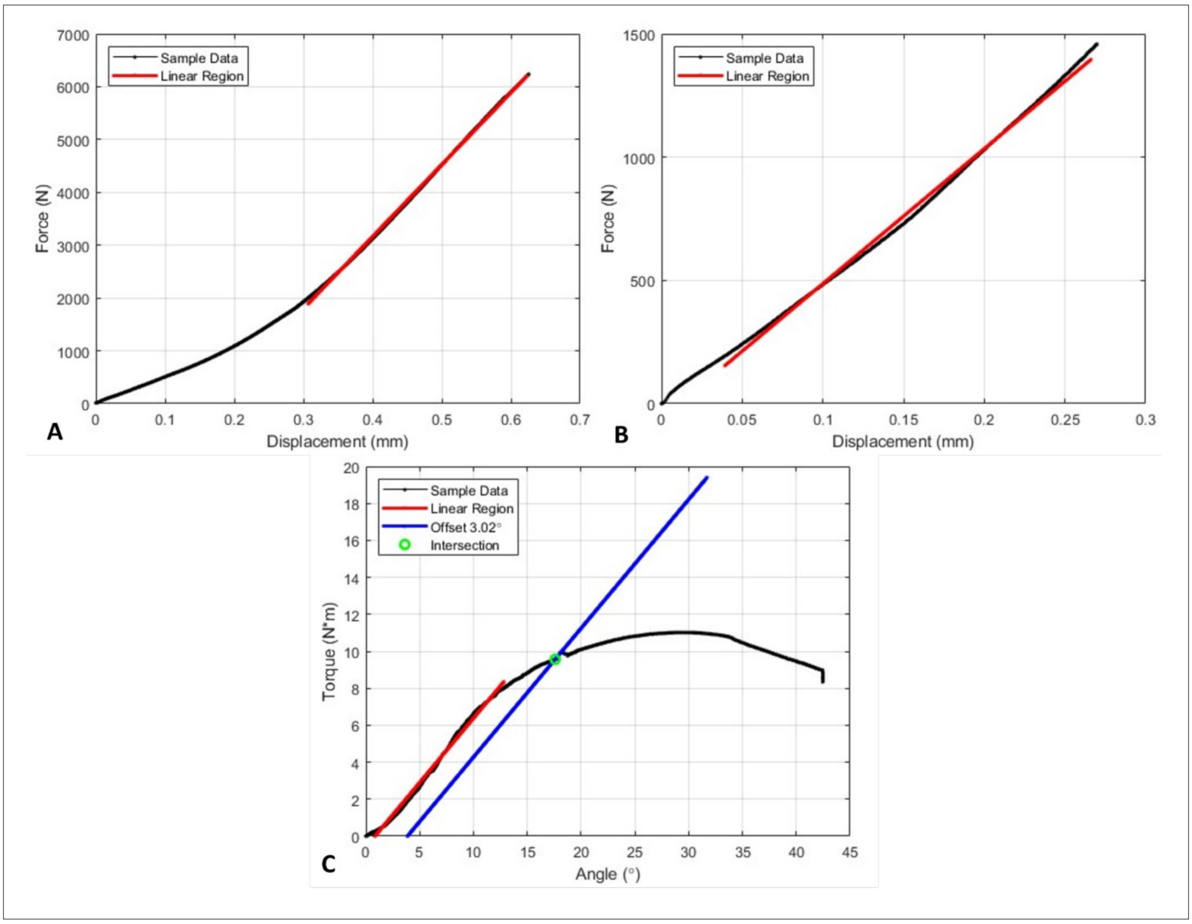

Figure S2. Representative force–displacement for compression (a) and shear (b) tests and torque–degree (c) curves of the samples (linear region was used to calculate stiffness, and intersection was used to determine the yield torque).

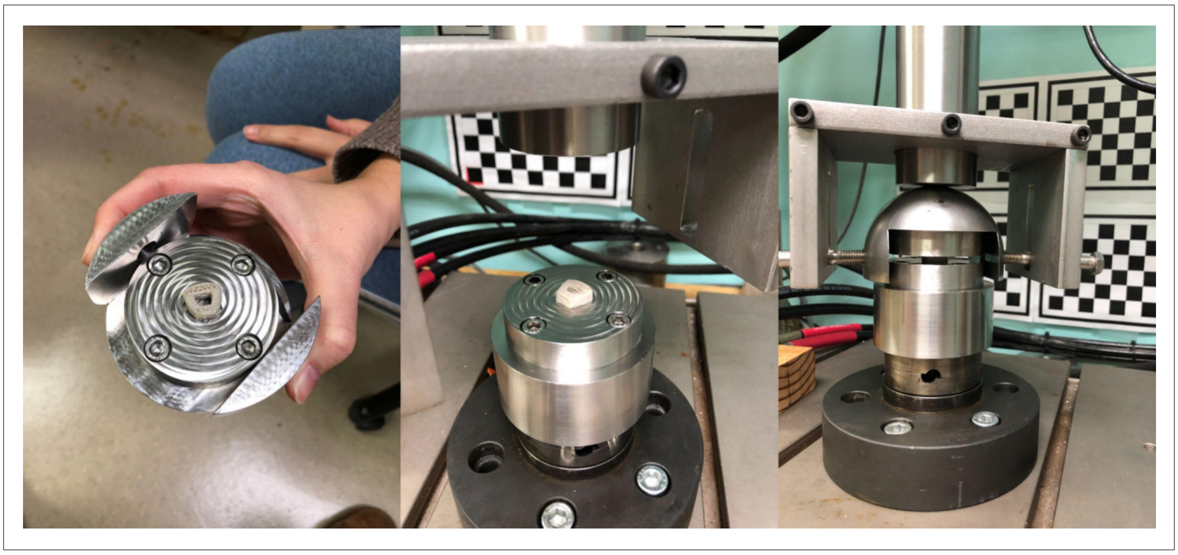

Figure S3. Torsion and compression (without the metal bracket) test set up according to ASTM F2077.[2]

References

1. Peck JH, Sing DC, Nagaraja S, Peck DG, Lotz JC, Dmitriev AE. Mechanical performance of cervical intervertebral body fusion devices: a systematic analysis of data submitted to the Food and Drug Administration. *J Biomech*. 2017;54:26-32. doi: 10.1016/j.jbiomech.2017.01.032

2. ASTM International. *Standard Test Methods for Intervertebral Body Fusion Devices*. PA, USA: ASTM; 2022. doi: 10.1520/F2077-22
